# Multiple *Plasmodium falciparum* drug resistance polymorphisms identified in a pregnant woman with severe malaria and a concomitant spontaneous abortion in Cross River, Nigeria, West Africa

**DOI:** 10.1186/s12936-022-04176-9

**Published:** 2022-06-03

**Authors:** Mary Aigbiremo Oboh, Fatou Faal, Oluwagbemisola Elizabeth Adeniji, Simon Correa, Anthony Uyimulam Amawu, Ekon Ogban, Eva Heinz, Grant Hughes, Martin M. Meremikwu, Alfred Amambua-Ngwa

**Affiliations:** 1 Medical Research Council Unit The Gambia, London School of Hygiene and Tropical Medicine, Fajara, The Gambia; 2 Department of Biomedical Sciences, Rochester Institute of Technology, Rochester, New York USA; 3 Malaria Genomic Epidemiology Network, Nigeria (MGENN), NIMR Lagos, Yaba, Nigeria; 4 Akpet Central Cottage General Hospital, Biase LGA, Akpet, Cross River State Nigeria; 5 Liverpool School of Tropical Medicine, Liverpool, UK; 6 University of Calabar Teaching Hospital, Calabar, Cross River State Nigeria

**Keywords:** *Plasmodium falciparum*, Severe malaria, Pregnant woman, Quinine, Single nucleotide polymorphism

## Abstract

**Background:**

The development of resistance by *Plasmodium falciparum* to anti-malarial drugs impedes any benefits of the drug. In addition, absence or delayed availability of current anti-malarial drugs in remote areas has the potential to results to parasite escape and continuous transmission.

**Case presentation:**

The case of a 29-year old pregnant woman from Biase Local Government Area in Cross River State Nigeria presenting with febrile illness and high body temperature of 38.7 °C was reported. She looked pale and vomited twice on arrival at the health facility. Her blood smear on the first day of hospitalization was positive for *P. falciparum* by RDT, microscopy (21,960 parasite/µl) and real-time PCR, with a PCV of 18%. She was treated with 600 mg intravenous quinine in 500 ml of 5% Dextrose/0.9% Saline 8-hourly for 24 h. On the second day of hospitalization, she complained of weakness, persistent high-grade fever and vaginal bleeding. A bulging amnion from an extended cervix was observed. Following venous blood collection for laboratory investigations, 600 µg of misoprostol was inserted into the posterior fornix of her vagina as part of her obstetric care. Parenteral quinine was discontinued, and she was given full therapeutic regimen of artemether-lumefantrine 80/480 mg tablets to be taken for 3 days beginning from the second day. Her blood samples on the second and third day of hospitalization remained positive for *P. falciparum* by all three diagnostic methods. Single nucleotide polymorphism (SNP) assay on all three *P. falciparum* isolates revealed the presence of variants associated with multiple drug resistant markers.

**Discussion:**

Infecting *P. falciparum* isolates may have been resistant to initial quinine treatment resulting from parasite cross-resistance with other quinoline associated resistant markers such as 86Y and 184 F.

**Conclusions:**

Therefore, the likely transmission of similarly resistant parasites in the study area calls for reinforcement of interventions and adherence to current World Health Organization guidelines in administering only approved drugs to individuals in order to mitigate parasite escape and eventual transmission to other susceptible individuals.

## Background

Pregnancy-associated malaria (PAM) is a common occurrence in women living in malaria endemic areas in tropical and sub-tropical regions of the world such as in Africa [1] PAM is defined as the detection of asexual parasite stages in peripheral or sequestered blood cells from a pregnant woman [1]. The recent world malaria report estimated that 33.2 million pregnancies occurred in the World Health organization (WHO) Africa region, out of which 11.6 million were exposed to malaria [2]. *Plasmodium falciparum* is the most implicated species in PAM and it results in variable complications both in the mother and the fetus [3], sometimes leading to maternal anaemia, spontaneous abortion, stillbirths, premature and low birth weight [4].

Primigravid women are more susceptible to *P. falciparum* infections than the multigravida, as a result of pregnancy-related suppression of immunity and their lack of parity-dependent malaria immunity acquired from exposure to multiple malaria infections in successive pregnancies [3, 5, 6]. Intermittent preventive treatment in pregnancy (IPTp) with the use of sulfadoxine-pyrimethamine (SP) in at least three monthly doses after the first trimester has been shown to significantly reduce the deleterious effects of malaria on the mother and fetus [7]. Nevertheless, the development of resistance to drugs by *P. falciparum* in some cases impedes and overshadows the benefits provided by IPTp-SP.

Parasite resistance to drug is one of the prevailing challenges in the fight against malaria in Africa and elsewhere in the world. Parasite develop resistance to anti-malarial drug principally when there is strong drug pressure on the parasite resulting in mutational changes as well as increase copy numbers in the parasite drug targets [8]. Both multi-drug resistance involving different molecular markers and cross resistance with drugs that have similar mode of action constitute a growing challenge in the control and containment of drug resistant genotypes. Thus, combating *P. falciparum* resistance to drug requires strategic planning, prompt action and concerted effort for sustainability. In this case report, we present a pregnant woman with severe *P. falciparum* infection, a spontaneous abortion and multiple multi drug resistant genotypes.

## Case presentation

In February 2021, a 29-year old pregnant woman visited the Akpet Central Cottage General Hospital in Biase local government area of Cross River State Nigeria. This hospital serves several rural communities in Nigeria’s Niger Delta region with high and perennial malaria transmission. On presentation, she complained of waist pain, chills, weakness, nausea, vomiting and high fever. Her estimated gestational age based on last menstrual period was 11–12 weeks, she has had 5 previous pregnancies with 4 live births and 1 abortion. Fever had started about 2 weeks before visiting the hospital for which she had only taken paracetamol before visiting the hospital.

She vomited twice on admission in addition to several vomits earlier that day. On examination, she was pale and moderately dehydrated with temperature of 38.7 °C, pulse of 105 beats/min, respiration rate of 32 breaths/min, and blood pressure of 80/40 mm Hg. Her lower abdomen (suprapubic region) was tender, vaginal examination revealed discharge of clear fluid and bulging membrane from the cervix. Obstetric ultrasound scan revealed the presence of a singleton active fetus with cephalic presentation and 105 beat per minutes. Initial provisional diagnosis was sepsis, secondary cervical incompetence and severe malaria, all of which could lead to irreversible miscarriage. Although she had a cerclage done for probable cervical incompetence in a previous pregnancy, the procedure did not prevent the loss of that earlier pregnancy which occurred at about the same gestational age as the current episode. Blood sample was drawn from her on arrival (and on the subsequent 2 days that she stayed in the hospital). Malaria rapid diagnostic test (mRDT) conducted using the *P. falciparum* histidine rich protein II test kit (SD Bioline, Abbot US) was positive for *P. falciparum* while light microscopy showed parasitaemia of 21,960 parasites/µl, 14,000 parasites/µl and 10,000 parasites/µl on day 1, 2 and 3, respectively. Thick blood films prepared with 10% Giemsa was used to estimate parasite density per 200 leukocytes assuming a white blood cell count of 8000 parasites/µl as detailed in a previous study [9]. She was treated with intravenous quinine 600 mg in 500 ml of 5% dextrose /0.9% saline 8-hourly for 24 h. She also received 10 mg intravenous diazepam, 1 L of 5% dextrose saline (DS) IV infusion, 1 g ceftriaxone IV daily for 48 h, 500 mg metronidazole IV 8-hourly for 48 h.

The next day, the patient complained of weakness and persistent high-grade fever (> 38.5 °C). Her packed cell volume (PCV) was evaluated to be 18%, which is lower than usual (33–38%) and consistent with the diagnosis of severe malaria. Later, in her second day of hospitalization, she complained of vaginal bleeding and closer examination revealed a bulging amnion from a widened cervix into the vaginal space. 600 µg of misoprostol was inserted into the posterior fornix of her vagina and she was taken into the delivery room for eventual expulsion which occurred around 03:00 h GMT the third day of her admission. Following expulsion, she was administered 1 ml of oxytocin and 1amp of IV vitamin K. She was given full therapeutic regimen of artemether-lumefantrine 80/480 mg tablets to be taken three days; and parenteral quinine was discontinued. She was also given fesolate (ferrous sulfate 65 mg elemental iron) tablets (twice daily) and folic acid (5 mg once daily). Her peripheral blood sample remained positive for malaria for the three days that she was on admission before absconding from the hospital.

Blood samples collected on the three consecutive days were preserved on filter papers as dried blood spots (DBS). DNA was subsequently extracted from this DBS and, *P. falciparum* confirmed using a real-time PCR targeting the varATS gene and an in-house conventional PCR that focused on the apical membrane protein 1 and circumsporozoite protein of *P*. *falciparum* (Oboh et al., unpublished).

Furthermore, high resolution melt (HRM) analysis for drug assay as described previously [10] using the Type IT master mix was employed to probe for molecular markers associated with resistance to the quinoline family of drug *(Pfmdr1*), sulfadoxine-pyrimethamine (Pfdhfr and *Pfdhps*) and chloroquine (*Pfcrt)*.

**Fig. 1:**
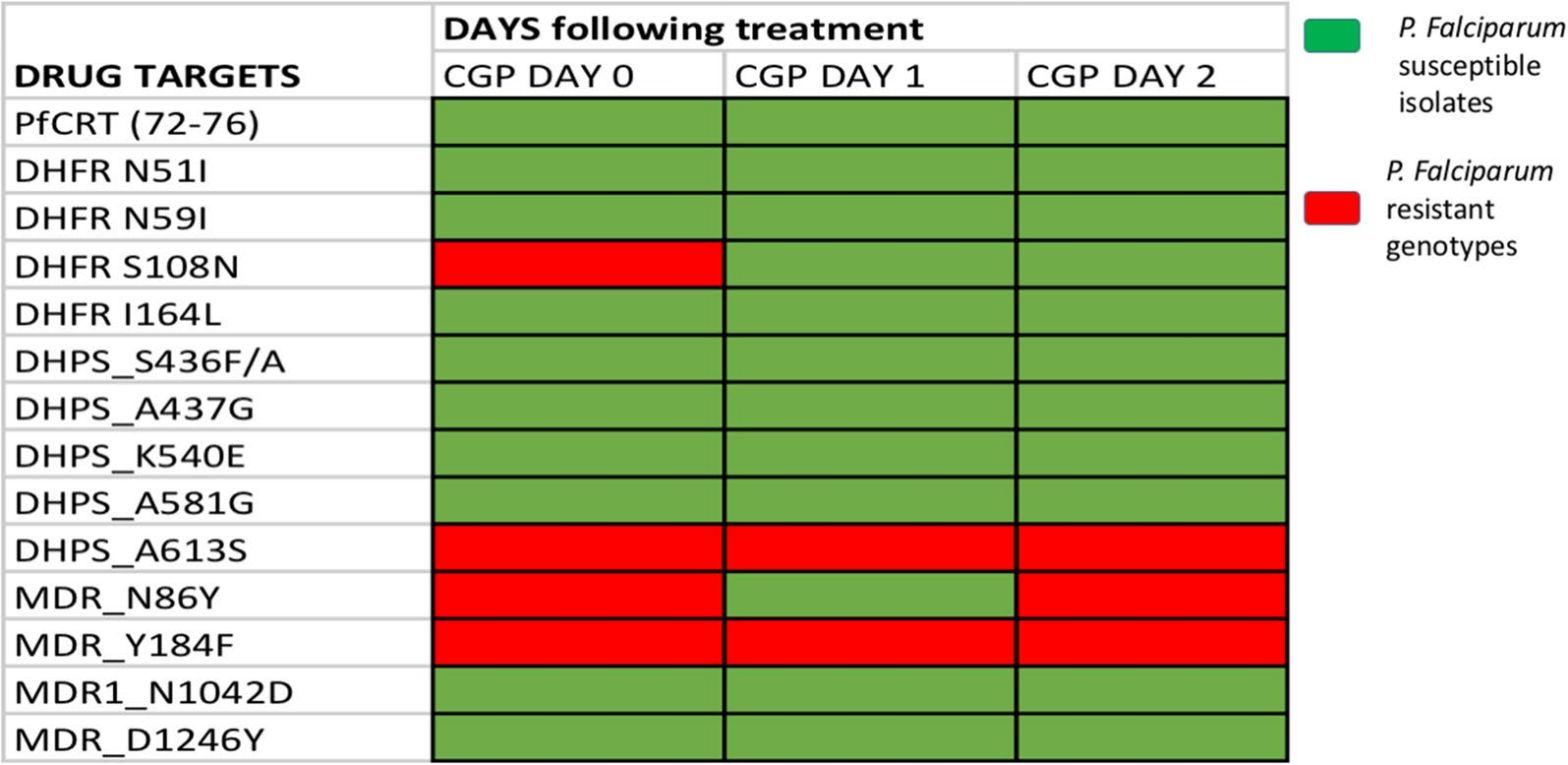
*Plasmodium falciparum* drug resistant genotypes from a pregnant woman with severe malaria

Resistance markers specifically associated with the quinoline family of drugs such as quinine (*Pfmdr* N86Y: day 1 and 3; *Pfmdr* Y184F all three isolates) were observed in the collected isolates (Fig. 1). Moreover, nucleotide substitution at codons associated with resistance to pyrimethamine (*Pfdhfr* S108N day 1 isolate) and sulfadoxine (*Pfdhps* A613S—all three isolates) were noted.

## Discussion

The effects of *P. falciparum* in pregnancy are often severe and affects both the fetus and the pregnant mother. Pregnancy associated malaria (PAM) is mostly aggravated in primigravidae who are immune-naïve, and are more vulnerable than the multigravidae with accumulated immunity from previous pregnancies and exposure to malaria infections.

Despite a history of cerclage suggestive of incompetent cervix, the severity of *P. falciparum* infection and the resultant severe anaemia (haematocrit of 18% and 17% PCV on day 2 and 3 of admission with the concomitant parasitaemia) could have contributed to the observed abortion. Her peripheral blood samples remained positive for malaria parasites during the three consecutive days of admission following mRDT, microscopy and quantitative PCR assay as described in an earlier study [11].

In order to ascertain that *P. falciparum* infection in her is an active infection, an in-house assay targeting *P*. *falciparum* apical membrane protein 1 and circumsporozoite protein were designed to amplify a 750 and 890 bp fragment (Oboh et al., unpublished), and in the consecutive isolates collected from the patient. This taken together with the microscopy result shows that mRDT positivity in her was not because of persistent antigenaemia, but current active infection.

Quinine is no longer recommended as treatment of choice for severe malaria since it was shown to be less effective in preventing deaths from severe malaria when compared to parenteral artesunate in a randomized comparative trial [12], but was used in this case as the attending physician preference in the absence of injection artesunate at that health facility during her period of hospitalization. To determine if persistent parasitaemia in the patient is associated with cross resistant in known molecular markers in the quinoline family of drugs, a panel of single nucleotide polymorphisms (SNPs) in anti-malarial drug resistance associated genes was assayed on the three isolates using high resolution melting analysis [13]. Resistance markers specifically associated with the quinoline family of drugs, such as quinine (*Pfmdr* N86Y: day 1 and 3; *Pfmdr* Y184F all three isolates) were observed in the collected isolates (Fig. 1). Moreover, nucleotide substitution at codons associated with resistance to pyrimethamine (*Pfdhfr* S108N day 1 isolate); sulfadoxine (*Pfdhps* A613S—all three isolates) were also observed. Nevertheless, it is not clear if the mutant genotypes detected in these isolates especially with regards to *Pfmdr1* is the result of secondary cross resistance resulting from the use of quinine as has been detected in a previous study [14]. Thus, it is probable that the infecting *P. falciparum* isolates may have been resistant to initial quinine treatment in this case. Although, whole genome sequencing of the samples obtained from the three consecutive days would have provided more detailed information, the importance of this finding cannot be undermined. Therefore, if similar parasites are freely circulating and perhaps in transmission in the study area, the patient, who absconded from the health facility before the treatment was completed could become a source of circulating more resistant infections back into the community. Although, the particular reason for her hospital escape could not be traced, it can be speculated to be somewhat associated with the socio-economic context. Medical aid to vulnerable group such as pregnant women and children in low income settings is a feasible way to prevent such from happening in the future.

## Data Availability

All data generated from this study has been embedded in this manuscript.

## Abbreviations

PAM: Pregnancy associated malaria
IPTp: Intermittent preventive treatment in pregnancy
SP: Sulfadoxine-pyrimethamine
IV: Intravenous
DS: Dextrose saline
PCV: Packed cell volume
GMT: Greenwich Meridian Time
RDT: Malaria rapid diagnostic test
